# miR-Let7A Controls the Cell Death and Tight Junction Density of Brain Endothelial Cells under High Glucose Condition

**DOI:** 10.1155/2017/6051874

**Published:** 2017-06-07

**Authors:** Juhyun Song, So Ra Yoon, Oh Yoen Kim

**Affiliations:** ^1^Department of Biomedical Sciences, Center for Creative Biomedical Scientists at Chonnam National University, Gwangju 61469, Republic of Korea; ^2^Department of Food Science and Nutrition, Dong-A University, Brain Busan 21 Project, Busan 49315, Republic of Korea

## Abstract

Hyperglycemia-induced stress in the brain of patients with diabetes triggers the disruption of blood-brain barrier (BBB), leading to diverse neurological diseases including stroke and dementia. Recently, the role of microRNA becomes an interest in the research for deciphering the mechanism of brain endothelial cell damage under hyperglycemia. Therefore, we investigated whether mircoRNA Let7A (miR-Let7A) controls the damage of brain endothelial (bEnd.3) cells against high glucose condition. Cell viability, cell death marker expressions (p-53, Bax, and cleaved poly ADP-ribose polymerase), the loss of tight junction proteins (ZO-1 and claudin-5), proinflammatory response (interleukin-6, tumor necrosis factor-*α*), inducible nitric oxide synthase, and nitrite production were confirmed using MTT, reverse transcription-PCR, quantitative-PCR, Western blotting, immunofluorescence, and Griess reagent assay. miR-Let7A overexpression significantly prevented cell death and loss of tight junction proteins and attenuated proinflammatory response and nitrite production in the bEnd.3 cells under high glucose condition. Taken together, we suggest that miR-Let7A may attenuate brain endothelial cell damage by controlling cell death signaling, loss of tight junction proteins, and proinflammatory response against high glucose stress. In the future, the manipulation of miR-Let7A may be a novel solution in controlling BBB disruption which leads to the central nervous system diseases.

## 1. Introduction

Blood-brain barrier (BBB) is a well-organized physiological structure composed of several cells such as brain endothelial cells, pericytes, and astrocytes from the central nervous system (CNS), which tightly controls the movement of cells and molecules between the blood and brain parenchyma [1]. Brain endothelial cells as a main component of BBB are linked together by the interactions between tight junction proteins such as claudins and occludin [2]. As an intact BBB is necessary for maintaining the microenvironment of the brain, the breakdown of BBB is associated with the occurrence of various diseases including stroke, dementia, and epilepsy [3, 4]. Several studies demonstrated that diabetes triggers the risk of neurological dysfunction due to the BBB disruption [5, 6]. High glucose condition in diabetes triggers the alteration of microvascular [7, 8] and neurovascular unit [6]. To control the microenvironment of BBB in diabetes, many researchers investigated how high glucose condition triggers the death of brain endothelial cells [9, 10] and the degradation of tight junction protein which is involved in the BBB permeability [11, 12].

MicroRNAs as nonprotein-coding RNAs with 21–25 nucleotides have been known to control various gene expressions at the posttranscriptional level by targeting with 3′-UTR of mRNA and play an important regulator of metabolic disease, thereby emerging as biomarkers and therapeutic targets [13, 14]. Among the microRNAs, microRNA-Let7A (miR-Let7A) is recently suggested as a therapeutic target for improvement of the pathological conditions related to metabolic diseases (i.e., cancer, diabetes, obesity, inflammation, vascular disease, etc.) [15–25]. It was known to be involved in cellular senescence, glucose metabolism, and inflammatory pathways [15–25]. In high glucose condition, miR-Let7A could control the cytokine production [21] and inflammatory mechanisms [25, 20]. However, the regulatory effects of miR-Let7A on metabolic status in associated with hyperglycemic condition were different among the studies: some reported the protective properties of miR-Let7A, but the others presented the negative properties [15–26]. As mentioned above, recent studies reported the involvement of microRNAs in the control of BBB disruption, but the mechanism is not fully understood. Furthermore, there are few studies on the role of miR-Let7A in cerebrovascular system including BBB under hyperglycemic condition. Therefore, the present study aimed to investigate the role of miR-Let7A in brain endothelial cells, for example, apoptosis, tight junction protein expression, and inflammatory response under high glucose condition, which may suggest that the manipulation of miR-Let7A would be a crucial issue for the protection of BBB disruption.

## 2. Materials and Methods

### 2.1. Cell Culture

Mouse brain endothelial cells (bEnd.3 cells) (ATCC Manassas, VA, USA) were cultured in Dulbecco Modified Eagle Medium (DMEM) (Gibco, Grand Island, NY, USA) which contained 0.45% glucose, 0.37% NaHCO_3_, 4 mM glutamine, 10% fetal bovine serum (FBS), 100 *μ*g/ml penicillin, and 100 *μ*g/ml streptomycin. bEnd.3 cells were cultured in a humidified incubator at 37°C with 5% CO_2_. The cells were treated with D-glucose (Sigma-Aldrich, St. Louis, MO, USA) at various concentrations (100 *μ*M, 10 mM, and 25 mM) for 24 hours.

### 2.2. 3-(4,5-Dimethylthiazol-2-yl)-2,5-diphenyltetrazolium Bromide (MTT) Assay

To examine the cell viability, we performed a 3-(4,5-dimethylthiazol-2-yl)-2,5-diphenyltetrazolium bromide (MTT) assay. After exposure to various concentrations of glucose, bEnd.3 cells (2 × 10^5^ cells/ml) were rinsed three times with PBS. Next, the culture medium was replaced with a serum-free medium, and 100 *μ*l of MTT (Sigma-Aldrich, St. Louis, MO, USA) solution (2 mg/ml in PBS) was treated to each well. After 1 hour 30 min of incubation, the medium was removed, and dimethyl sulfoxide (DMSO) 0.1% was added to form a solution with the formazan reaction product. The supernatant from each well was measured using an ELISA reader at a wavelength of 570 nm. All experiments were repeated three times. Cell viabilities are calculated relative to nontreated controls, whose cell viabilities are considered to be 100%.

### 2.3. MicroRNA Transfection

miR-Let7A was purchased from Ambion (Austin, TX, USA) and followed the manufacturer's protocol. Two kinds of miR-Let7A were used in this study; Let-7A mimic (catalogue number, 4464066; assay ID, MC10050) and Let-7A inhibitor (catalogue number, 4464084; assay ID, MH 10050). The expression and inhibition levels were validated with negative (catalogue number, 4464058) and positive control miRNA (catalogue number, 4464062). bEnd.3 cells were cultured for 2 days and were then treated with miR-Let-7A and miRNA negative control for each group.

### 2.4. Western Blot Analysis

bEnd.3 cells were washed with PBS and collected. The cell pellets were lysed with cold RIPA 20 min at 4°C to produce whole-cell extracts. Protein (25 *μ*g) in cells was separated on a 12% SDS-polyacrylamide gel and transferred onto a polyvinylidene difluoride membrane. After blocking with skim milk prepared in tris-buffered saline—Tween (TBST) (20 nM tris, pH 7.2, 150 mM NaCl, 0.1% Tween 20) for 1 hour and 30 min at room temperature, immunoblots were incubated for 14 hours at 4°C with primary antibodies that detect cleaved poly ADP-ribose polymerase (PARP) (1 : 1000, Abcam, Cambridge, MA, USA), claudin 5 (CLD5) (1 : 1000, Cell Signaling, Danvers, MA, USA), or *β*-actin (1 : 1000; Millipore, Billerica, MA, USA). Blots were then incubated with specific secondary antibodies (Abcam, Cambridge, MA, USA) for 2 hours at room temperature. Blots were visualized by ECL solution (Millipore, Billerica, MA, USA).

### 2.5. Reverse Transcription Polymerase Chain Reaction (RT-PCR)

RNA in bEnd.3 cells was extracted using Trizol Reagent (Gibco, Grand Island, NY, USA). Reverse transcription polymerase chain reaction (RT-PCR) was conducted by using Invitrogen One step III™ Reverse Transcription PCR kit (Invitrogen, Carlsbad, CA, USA). PCR was performed following thermal cycling conditions: 95°C for 10 min; 40 cycles of denaturing at 95°C for 15 seconds, annealing at 60.5°C for 30 seconds, and elongation at 72°C for 30 seconds; final extension at 72°C for 5 minutes; and holding at 4°C. PCR was performed using the following primers (5′ to 3′): tumor necrosis factor (TNF)-*α* (F); CGT CAG CCG ATT TGC TAT CT, (R); CGG ACT CCG CAA AGT CTA AG; interleukin (IL)-6 (F); GTT GCC TTC TTG GGA CTG AT, (R); CTG GCT TTG TCT TTC TTG TTA T; CLD5 (F); CTG CTG GTT CGC CAA CAT T, (R); TGC GAC ACG GGC ACA G; inducible nitric oxide synthase (iNOS) (F); GGG AAT CTT GGA GCG AGT TG, (R); GTG AGG GCT TGG CTG AGT GA, Bax (F); AAG AAG CTG AGC GAG TGT, (R); GGA GGA AGT CCA ATG TC, p-53 (F); GAG TGT TCC GTG TAT GGC AC, (R); GAT GCC TTG GAT GAT GGT C, ZO-1 CAG CCG GTC ACG ATC TCC T, (R); TCC GGA GAC TGC CAT TGC, GAPDH (F); GAC AAG CTT CCC GTT CTC AG, (R); GAG TCA ACG GAT TTG GTC GT. PCR products were electrophoresed in 1% agarose gels and stained with mango blue. All samples were normalized with GAPDH.

### 2.6. TaqMan Assay for miRNA

To analyze the level of miR-Let7A, reverse transcription was performed using the Taqman microRNA reverse transcription kit (Applied Biosystems, Waltham, Massachusetts, USA) with 10 g RNA. The PCR reactions were conducted as per the manufacturer's protocol to quantitate the level of miRNA Let7A using Taqman Universal PCR Master Mix, No Amp Erase UNG (Applied Biosystems, Waltham, Massachusetts, USA) and Taqman microRNA assay (Applied Biosystems, Waltham, Massachusetts, USA) for miR-Let7A. PCR amplification was conducted in Takara Real Time PCR (Takara, Tokyo, Japan) at 95°C for 10 minutes, followed by 40 cycles at 95°C for 15 seconds, and 60°C for 60 seconds. The PCR incubation profile was extended to 40 cycles for miR-Let7A. The differential level was calculated using the ΔΔCt method. The level of miRNA Let7A was represented as a relative quantity (RQ) normalized to U6. The PCR reactions were conducted three times.

### 2.7. Quantitative Real-Time PCR

Total cellular RNA was extracted from the cells using Trizol Reagent (Invitrogen, Carlsbad, CA, USA) according to the manufacturer's instructions. Poly (A) was added using poly (A) polymerase (Ambion, Austin, TX, USA). One Step SYBR® Prime Script™ RT-PCR Kit II (Takara, Japan) was used to conduct qPCR. PCR was performed using the following primers (5′ to 3′): ZO-1 CAG CCG GTC ACG ATC TCC T, (R); TCC GGA GAC TGC CAT TGC, TNF-*α* (F); CGT CAG CCG ATT TGC TAT CT, (R); CGG ACT CCG CAA AGT CTA AG, iNOS (F); GGG AAT CTT GGA GCG AGT TG, (R); GTG AGG GCT TGG CTG AGT GA. The expression of each factors was assessed using an ABI prism 7500 Real-Time PCR System (Life Technologies Corporation, CA, USA) and analyzed with comparative Ct quantification. *β*-actin was amplified as an internal control. The values were presented by relative quantity (RQ). All experiments were repeated three times.

### 2.8. Immunocytochemistry

bEnd.3 cells were washed thrice with PBS and were permeabilized for 20 minutes. bEnd.3 cells were incubated with the primary antibodies for 12 hours at 4°C. The following primary antibodies were used: anti-rabbit CLD5 (1 : 500, Cell Signaling, Danvers, MA, USA) and anti-rabbit cleaved PARP (1 : 500, Abcam, Cambridge, MA, USA). After 16 hours incubation, bEnd.3 cells were washed three times with PBS and incubated with each specific secondary antibody for 1 hour at room temperature. bEnd.3 cells were counterstained with 1 *μ*g/ml 4′,6-diamidino-2-phenylindole (DAPI, 1 : 100, Invitrogen, Carlsbad, CA, USA) for 5 minutes at room temperature. Images were obtained using confocal microscope (Carl Zeiss, Thornwood, NY, USA). Cells in three randomly selected fields were measured for immunodensity using ImageJ software (ImageJ, Madison, Wisconsin, USA).

### 2.9. Determination of Nitrite Production

bEnd.3 cells were seeded onto 96-well plates at density of 5 × 10^4^ cells/well and pretreated with D-glucose (25 mM), Let7A mimic or Let7A inhibitor. The supernatants were collected and assessed for nitrite production using Griess reagent (Sigma-Aldrich, St. Louis, MO, USA). The Griess reagents (100 *μ*l) were added and incubated for 30 minutes at room temperature. The absorbance of supernatants was measured at 540 nm using the ELISA reader (Versamax Molecular Devices, Hampton, NH, USA).

### 2.10. Statistical Analysis

Statistical analysis was conducted by SPSS 23.0 software (IBM Corp., Armonk, NY, USA). The results are expressed as the mean ± standard deviations (SD). Statistical analyses were performed using one-way analysis of variance (ANOVA) followed by Bonferroni post hoc multiple comparison. Each experiment included at least 3 replicates per condition. A *P* value less than 0.05 was considered statistically significant.

## 3. Results

### 3.1. High Glucose Condition Is Associated with Cell Death of bEnd.3 Cells under High Glucose In Vitro Condition

**Figure 1:**
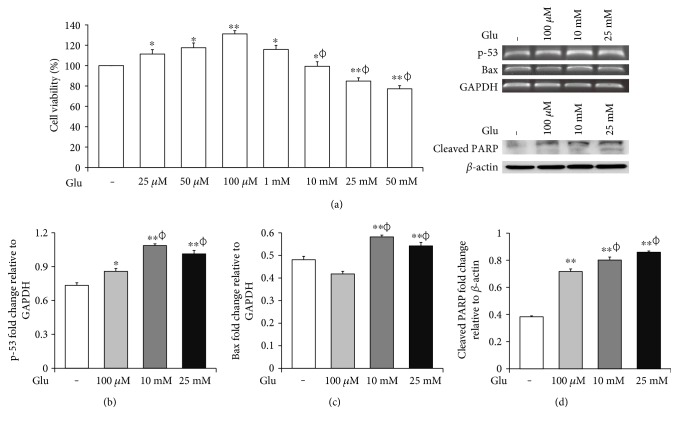
High glucose triggers cell death of bEnd.3 cells under high glucose in vitro condition. Cell viability was tested by the MTT assay (a). The mRNA levels of p-53 (a) and Bax (b) were measured by reverse transcription PCR, and protein levels of cleaved PARP (c) were measured by Western blot analysis. The results are expressed as the mean ± standard deviations (SD). Each experiment included at least 3 replicates per condition. ^∗^*p* < 0.05 and ^∗∗^*p* < 0.001 compared with nontreated control cells; *^ϕ^p* < 0.05 compared with 100 *μ*M glucose-treated cells. Glu: D-glucose treatment for 24 hours; PARP: poly ADP-ribose polymerase.

To assess the cell death of bEnd.3 cells under high glucose condition, we performed MTT (Figure 1(a)), RT-PCR (p-53 and Bax, Figures 1(b) and 1(c)) and Western blot (cleaved PARP, Figure 1(d)) analyses. The cell viability was dose dependently increased by 25, 50, and 100 *μ*M of glucose and then gradually decreased at 10, 25, and 50 mM of glucose. Particularly, the cell viabilities exposed at 25 and 50 mM of glucose were significantly reduced than those of nontreated control cells (Figure 1(a)). Therefore, we selected 100 *μ*M, 10 mM, and 25 mM of glucose for further experiment. mRNA levels of p-53 were significantly increased by glucose treatment (100 *μ*M, 10 mM, and 25 mM) (Figure 1(b)), and those of Bax were also increased by glucose treatment particularly at concentrations of 10 mM and 25 mM (Figure 1(c)). In addition, cleaved PARP protein levels were significantly and gradually increased by glucose treatment (100 *μ*M, 10 mM, and 25 mM) (Figure 1(d)).

### 3.2. High Glucose Condition Is Associated with the Loss of Tight Junction Integrity in bEnd.3 Cells

**Figure 2:**
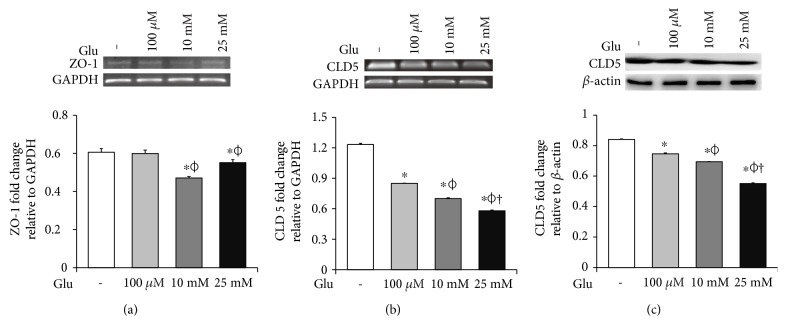
High glucose condition aggravates the loss of tight junction proteins in bEnd.3 cells. The mRNA levels of ZO-1 (a) and CLD5 (b) were measured by reverse transcription PCR, and protein levels of CLD5 (c) were measured by Western blot analysis. The results are expressed as the mean ± standard deviations (SD). Each experiment included at least 3 replicates per condition. ^∗^*p* < 0.05 compared with nontreated control cells; *^ϕ^p* < 0.05 compared with 100 *μ*M glucose-treated cells; ^†^*p* < 0.05 compared with 10 mM glucose-treated cells. Glu: D-glucose treatment for 24 hours; CLD5: claudin 5.

To examine the change of tight junction-related protein expression in bEnd.3 cells under high glucose condition, we conducted RT-PCR (ZO-1 and CLD5, Figures 2(a) and 2(b)) and Western blot analyses (CLD5, Figure 2(c)). The mRNA levels of ZO-1 in bEnd.3 cells were significantly attenuated by the treatment of glucose (10 mM and 25 mM) (Figure 2(a)). The mRNA levels of CLD5 were markedly and dose dependently decreased by glucose treatment (Figure 2(b)). In addition, protein levels of CLD5 were also dose dependently decreased by glucose treatment (Figure 2(c)).

### 3.3. High Glucose Condition Is Associated with Production of Proinflammatory Cytokines and iNOS in bEnd.3 Cells

**Figure 3:**
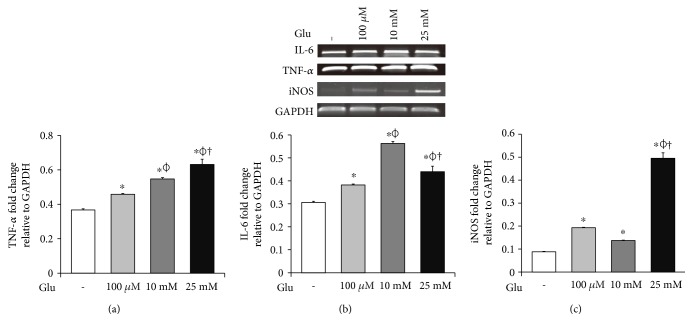
High glucose produces proinflammatory cytokines and iNOS in bEnd.3 cells. The mRNA levels of TNF-*α* (a), IL-6 (b), and iNOS (c) were measured by reverse transcription PCR. The results are expressed as the mean ± standard deviations (SD). Each experiment included at least 3 replicates per condition. ^∗^*p* < 0.05 compared with nontreated control cells; *^ϕ^p* < 0.05 compared with 100 *μ*M glucose-treated cells; ^†^*p* < 0.05 compared with 10 mM glucose-treated cells. Glu: D-glucose treatment for 24 hours; IL-6: interleukin-6; iNOS: inducible nitric oxide synthase; TNF-*α*: tumor necrosis factor-*α.*

To investigate the mRNA expression of proinflammatory cytokines and iNOS in the bEnd.3 cells under high glucose condition, we conducted RT-PCR (Figures 3(a), 3(b), and 3(c)). The mRNA levels of TNF-*α* were dose dependently increased by the treatment of glucose (Figure 3(a)). IL-6 mRNA levels were also significantly increased by the glucose treatment (Figure 3(b)). In addition, mRNA levels of iNOS were significantly increased by the glucose treatment: particularly, marked increase was observed at 25 mM of glucose (Figure 3(c)).

### 3.4. High Glucose Condition Is Involved in the Downregulation of miR-Let7A Expression in the (bEnd.3) Cells

**Figure 4:**
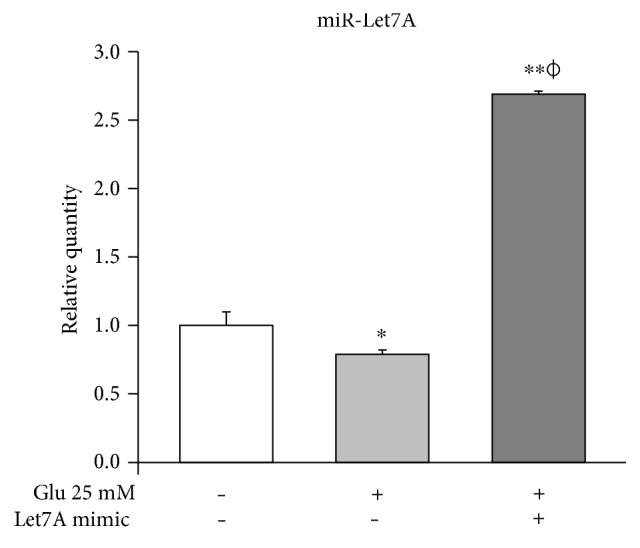
miR-Let-7A expression in the (bEnd.3) cells under high glucose condition. Expression of miR-Let7A was measured in all groups using TaqMan real-time PCR. The results are expressed as the mean ± standard deviations (SD). Each experiment included at least 3 replicates per condition. ^∗^*p* < 0.05 and ^∗∗^*p* < 0.001 compared with nontreated control cells; *^ϕ^p* < 0.05 compared with 25 mM glucose-treated cells.

Taqman assay was performed to compare the miR-Let7A expression level among the nontreated cells, 25 mM glucose only-treated cells, and Let7A mimic overexpressed and 25 mM glucose-treated cells. As shown in Figure 4, we found that miR-Let7A expression level in the 25 mM glucose only-treated cells was significantly lower than that in the nontreated cells. On the other hand, miR-Let7A expression level in the Let7A mimic overexpressed and 25 mM glucose-treated cells were markedly increased (more than 2.5-fold) compared with the nontreated cells or the 25 mM glucose only-treated cells. This result may show that miR-Let7A expression in the brain endothelial cells was downregulated under high glucose condition.

### 3.5. miR-Let7A Is Involved in the Regulation of Cell Death and Tight Junction Protein in bEnd.3 Cells under High Glucose In Vitro Condition

**Figure 5:**
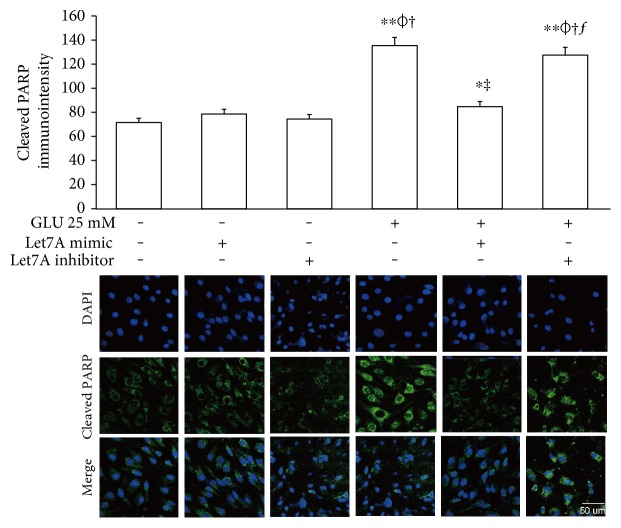
miR-Let7A attenuates the expression of cleaved PARP, a cell death marker in bEnd.3 cells under high glucose in vitro condition. To confirm the effect of miR-Let7A on the expression of cleaved PARP in high glucose-treated bEnd.3 cells, immunostaining was performed. Cells in three randomly selected fields were measured for immunodensity using ImageJ software (ImageJ, Madison, Wisconsin, USA). ^∗^*p* < 0.05 and ^∗∗^*p* < 0.001 compared with nontreated control cells; *^ϕ^p* < 0.05 compared with Let7A mimic overexpressed control cells; ^†^*p* < 0.05 compared with Let7A inhibitor overexpressed control cells; ^‡^*p* < 0.05 compared with 25 mM glucose-treated cells; ^ƒ^*p* < 0.05 compared with 25 mM glucose-treated and Let7A-overexpressed cells. Scale bar: 50 *μ*m, 4′,6-diamidino-2-phenylindole (DAPI): blue, cleaved PARP: green, Glu: D-glucose treatment for 24 hours, Let7A mimic: Let7A mimic pretreatment for 48 hrs, Let7A inhibitor: anti-Let7A pretreatment for 48 hrs, and PARP: poly ADP-ribose polymerase.

**Figure 6:**
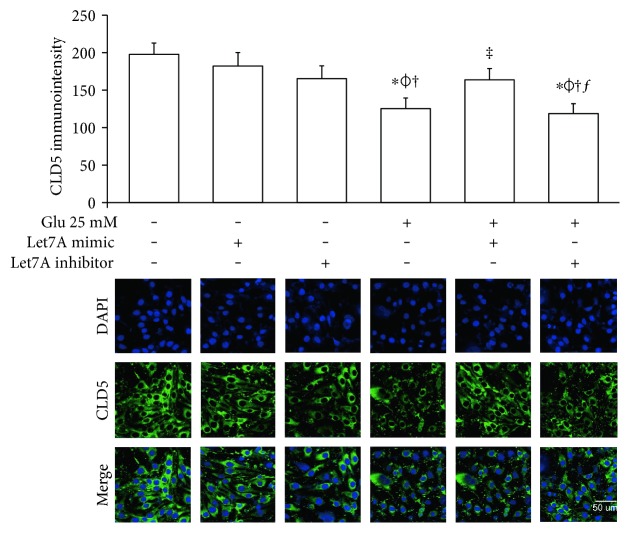
miR-Let7A ameliorates the expression of CLD5, a tight junction protein in bEnd.3 cells under high glucose in vitro condition. To confirm the effect of miR-Let7A on the expression of CLD5 in high glucose-treated bEnd.3 cells, immunostaining was performed. Cells in three randomly selected fields were measured for immunodensity using ImageJ software (ImageJ, Madison, Wisconsin, USA). ^∗^*p* < 0.05 compared with nontreated control cells; *^ϕ^p* < 0.05 compared with Let7A mimic overexpressed control cells; ^†^*p* < 0.05 compared with Let7A inhibitor overexpressed control cells; ^‡^*p* < 0.05 compared with 25 mM glucose-treated cells; ^ƒ^*p* < 0.05 compared with 25 mM glucose-treated and Let7A-overexpressed cells. Scale bar: 50 *μ*m, 4′,6-diamidino-2-phenylindole (DAPI): blue, cleaved PARP: green, Glu: D-glucose treatment for 24 hours, Let7A mimic: Let7A mimic pretreatment for 48 hrs, Let7A inhibitor: anti-Let7A pretreatment for 48 hrs, and CLD5: claudin 5.

**Figure 7:**
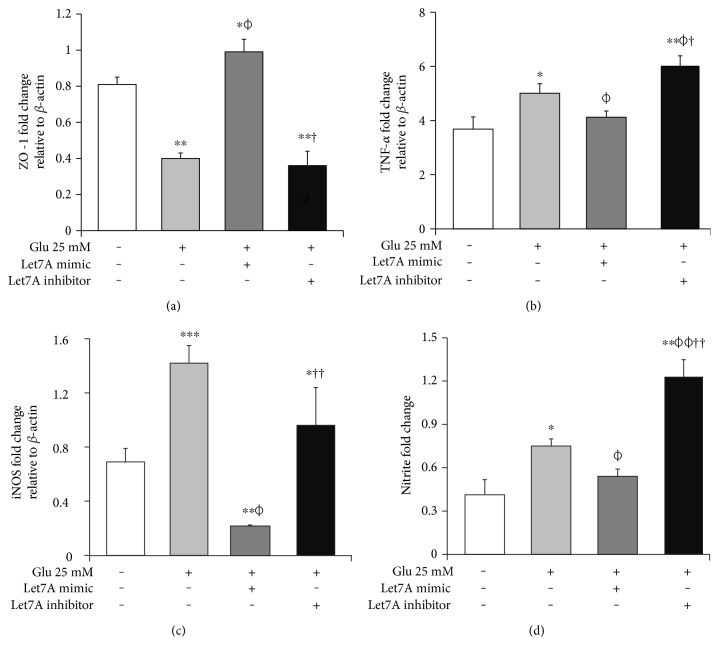
miR-Let7A modulates the expressions of ZO-1, TNF-*α*, and iNOS and nitrite production in bEnd.3 cells under high glucose in vitro condition. The mRNA levels of ZO-1 (a), TNF-*α* (b), and iNOS (b) were measured by quantitative real-time PCR. The production of nitrite (d) was detected by Griess reagent assay. The results are expressed as the mean ± standard deviations (SD). Each experiment included at least 3 replicates per condition. ^∗^*p* < 0.05 and ^∗∗^*p* < 0.001 compared with nontreated control cells; *^ϕ^p* < 0.05 compared with Let7A mimic overexpressed control cells; ^†^*p* < 0.05 compared with Let7A inhibitor overexpressed control cells; ^‡^*p* < 0.05 compared with 25 mM glucose-treated cells; ^ƒ^*p* < 0.05 compared with 25 mM glucose-treated and Let7A-overexpressed cells. Glu: D-glucose treatment for 24 hours, Let7A mimic: Let7A mimic pretreatment for 48 hrs, Let7A inhibitor: anti-Let7A pretreatment for 48 hrs, iNOS: inducible nitric oxide synthase, and TNF-*α*: tumor necrosis factor-*α.*

To confirm whether miR-Let7A is involved in the regulation of cell death and tight junction protein of bEnd.3 cells under high glucose condition, we performed immunofluorescence analysis for cleaved PARP and CLD5 with immunointensity calculation (Figures 5 and 6) and qPCR for ZO-1 mRNA expression (Figure 7(a)). Cleaved PARP was evidently increased by the treatment of 25 mM glucose. Interestingly, translocation of cleaved PARP into the nucleus was also observed in the glucose-treated bEnd.3 cells. On the other hand, miR-Let7A overexpression markedly attenuated the expression of cleaved PARP as well as its translocation into the nucleus in the glucose-treated bEnd.3 cells (Figure 5). Decreased CLD5 level under high glucose condition was significantly recovered by the overexpression of miR-Let7A (Figure 6). The mRNA levels of ZO-1 were significantly reduced by the treatment of 25 mM glucose compared with nontreated cells. The reduced mRNA levels of ZO-1 under high glucose condition were significantly recovered by the overexpression of miR-Let7A (Figure 7(a)). On the other hand, high glucose-induced alterations of cleaved PARP, CLD5, and ZO-1 were still retained when treated with miR-Let7A inhibitor (Figures 5 and 6).

### 3.6. miR-Let7A Attenuates the mRNA Expression of TNF-*α*, iNOS, and Nitrite Production in bEnd.3 Cells under High Glucose In Vitro Condition

To confirm whether miR-Let7A is involved in the regulation of proinflammatory cytokine and immune responses in the bEnd.3 cells under high glucose condition, we performed qPCR analysis for mRNA expressions of TNF-*α*, iNOS, and Griess reagent assay for nitrite production (Figures 7(b), 7(c), and 7(d)). mRNA levels of TNF-*α* were significantly increased under high glucose condition but significantly attenuated by miR-Let7A overexpression (Figure 7(b)). mRNA levels of iNOS was markedly increased under high glucose condition but significantly and greatly attenuated by miR-Let7A overexpression (Figure 7(c)). On the other hand, high glucose-induced increases of TNF-*α* and iNOS were still retained when treated with miR-Let7A inhibitor (Figures 7(b) and 7(c)). Nitrite production in the cells treated with 25 mM glucose was about 2 times higher than that in the nontreated cells. On the other hand, increased production of nitrite in the glucose-treated bEnd.3 cell was significantly reduced by miR-Let7A overexpression but markedly increased by the treatment of Let7A inhibitor (Figure 7(d)).

## 4. Discussion

The present study shows that miR-Let7A significantly prevented cell death and loss of tight junction proteins and attenuated proinflammatory response in the bEnd.3 cells under high glucose in vitro condition. It suggests that the manipulation of miR-Let7A may be a novel solution in controlling BBB disruption which leads to the CNS diseases.

Brain endothelial cells are the main cellular element of BBB that is necessary for CNS homeostasis [27]. They constitute the multiple network of vessels which transport nutrients and gases throughout the brain [28] and form the metabolic barrier [27, 29]. Brain endothelial cells are interconnected by complicated tight junctions between lateral plasma membranes [30]. The integrity of tight junctions was reported to be responsible for the brain endothelial permeability [31]; thus, the loss of tight junction proteins may cause BBB disruption, leading to the various CNS diseases [32–34]. Especially, diabetes is emerging as a critical issue in BBB breakdown [35, 36], suggesting that high glucose could damage the cells and attenuate the integrity of tight junction [37–39]. In this present study, we confirmed that high glucose activates mRNA expressions of p-53 and Bax and protein expression of cleaved PARP which indicates the activation of cell death signaling [35, 40, 41]. We also observed reduced mRNA expressions of ZO-1 and CLD5 and protein expression of CLD5 which present the loss of tight junction proteins in brain endothelial cells [32, 33, 36, 42].

Recent studies suggested that posttranscriptional gene regulation via microRNAs in brain endothelial cells may alleviate neuropathology of CNS diseases [43–50]. Of these miRNAs, miR-Let7A was reported to remarkably downregulate proinflammatory cytokines in neuroinflammation conditions [50] and moreover to support brain endothelial barrier function including increases of monocyte cell adhesion and migration [50]. In the present study, we observed that miR-Let7A expression in the brain endothelial cells was downregulated under high glucose condition, but miR-Let7A overexpression significantly attenuated proinflammatory cytokine production (i.e., TNF-*α*) [23], suppressed the increased expression of cleaved PARP and its translocation into the nucleus which indicate cell death signaling [51], and recovered the loss of tight junction proteins (i.e., CLD5 and ZO-1) in bEnd.3 cells [32, 33, 36, 42] under high glucose condition. In addition, we found that nitrite production and iNOS mRNA expression under high glucose condition [52] were significantly suppressed by miR-Let7A overexpression. From these results, we assume that miR-Let7A significantly attenuated the disruption of BBB and the cell death of brain endothelial cells. It may indicate that miR-Let7A plays a beneficial role against the loss of tight junction proteins, cell death signaling, and proinflammatory response in the brain endothelial cells under high glycemic condition.

This study has limitations. Animal study (i.e., knockout or knockin mouse model for miR-Let7A) together with in vitro experiment would be more supportive for the conclusion. Further study with animal model is needed to elucidate the role of miR-Let7A in brain endothelial system under hyperglycemic condition. Second, this study used nontreated cells and high glucose-treated cells as “control” based on the previous reports [16, 21–24], but using control mimic (i.e., *C.* elegans miR-2 or any other unrelated miR) in the control cells would be meaningful to elucidate the sole effect of miR-Let7A on brain endothelial cells in the future.

Despite the study limitations, we highlight three points in this study. First, high glucose aggravates the loss of tight junction proteins and cell death. Second, miR-Let7A contributes to the maintenance of tight junction integrity in spite of high glucose stress. Finally, miR-Let7A alleviates the apoptosis of brain endothelial cells under high glucose in vitro condition. Thus, we suggest that the manipulation of miR-Let7A would ameliorate the disruption of BBB by protecting brain endothelial cells in hyperglycemia condition.

## 5. Conclusions

From this study, we suggest that miR-Let7A could attenuate the damage of brain endothelial cells by controlling cell death signaling, loss of tight junction proteins, and proinflammatory response against high glucose stress. Furthermore, the manipulation of miR-Let7A may be a novel solution in controlling BBB disruption which leads to the CNS diseases.
